# Expression of concern: Investigation of the biological activity, mechanical properties and wound healing application of a novel scaffold based on lignin–agarose hydrogel and silk fibroin embedded zinc chromite nanoparticles

**DOI:** 10.1039/d4ra90104e

**Published:** 2024-09-19

**Authors:** Reza Eivazzadeh-Keihan, Hooman Aghamirza Moghim Aliabadi, Fateme Radinekiyan, Mohammad Sobhani, Farzane khalili, Ali Maleki, Hamid Madanchi, Mohammad Mahdavi, Ahmed Esmail Shalan

**Affiliations:** a Catalysts and Organic Synthesis Research Laboratory, Department of Chemistry, Iran University of Science and Technology Tehran 16846-13114 Iran maleki@iust.ac.ir +98-21-73021584 +98-21-73228313; b Faculty of Chemistry, K. N. Toosi University of Technology Tehran Iran; c Protein Chemistry Laboratory, Department of Medical Biotechnology, Biotechnology Research Center, Pasteur Institute of Iran Tehran Iran; d Department of Biotechnology, School of Medicine, Semnan University of Medical Sciences Semnan Iran hamidmadanchi@yahoo.com; e Drug Design and Bioinformatics Unit, Department of Medical Biotechnology, Biotechnology Research Center, Pasteur Institute of Iran Tehran Iran; f Endocrinology and Metabolism Research Center, Endocrinology and Metabolism Clinical Sciences Institute, Tehran University of Medical Sciences Tehran Iran; g BCMaterials, Basque Center for Materials, Applications and Nanostructures, Martina Casiano, UPV/EHU Science Park Barrio Sarriena s/n Leioa 48940 Spain; h Central Metallurgical Research and Development Institute (CMRDI) P. O. Box 87, Helwan Cairo 11421 Egypt a.shalan133@gmail.com

## Abstract

Expression of concern for ‘Investigation of the biological activity, mechanical properties and wound healing application of a novel scaffold based on lignin–agarose hydrogel and silk fibroin embedded zinc chromite nanoparticles’ by Reza Eivazzadeh-Keihan *et al.*, *RSC Adv.*, 2021, **11**, 17914–17923, https://doi.org/10.1039/D1RA01300A.

The Royal Society of Chemistry is publishing this expression of concern in order to alert readers that concerns have been raised regarding the reliability of the MTT plate image in Fig. 4B, the wound healing images in Fig 5B and the hemolysis plate image in Fig. 6B. An investigation is underway, and an expression of concern will continue to be associated with the article until a final outcome is reached.

Laura Fisher

13th September 2024

Executive Editor, *RSC Advances*

